# Does evolutionary innovation in pharyngeal jaws lead to rapid lineage diversification in labrid fishes?

**DOI:** 10.1186/1471-2148-9-255

**Published:** 2009-10-22

**Authors:** Michael E Alfaro, Chad D Brock, Barbara L Banbury, Peter C Wainwright

**Affiliations:** 1 University of California, Los Angeles 621 Charles E. Young Drive South, 5217 Life Sciences Building Los Angeles, CA 90095-1606, USA; 2 Washington State University Washington State University PO Box 644236. Pullman, WA 99164-4236, USA; 3 University of California, Davis, Department of Evolution and Ecology, University of California, One Shields Ave., Davis, CA 95616, USA

## Abstract

**Background:**

Major modifications to the pharyngeal jaw apparatus are widely regarded as a recurring evolutionary key innovation that has enabled adaptive radiation in many species-rich clades of percomorph fishes. However one of the central predictions of this hypothesis, that the acquisition of a modified pharyngeal jaw apparatus will be positively correlated with explosive lineage diversification, has never been tested. We applied comparative methods to a new time-calibrated phylogeny of labrid fishes to test whether diversification rates shifted at two scales where major pharyngeal jaw innovations have evolved: across all of Labridae and within the subclade of parrotfishes.

**Results:**

Diversification patterns within early labrids did not reflect rapid initial radiation. Much of modern labrid diversity stems from two recent rapid diversification events; one within julidine fishes and the other with the origin of the most species-rich clade of reef-associated parrotfishes. A secondary pharyngeal jaw innovation was correlated with rapid diversification within the parrotfishes. However diversification rate shifts within parrotfishes are more strongly correlated with the evolution of extreme dichromatism than with pharyngeal jaw modifications.

**Conclusion:**

The temporal lag between pharyngeal jaw modifications and changes in diversification rates casts doubt on the key innovation hypothesis as a simple explanation for much of the richness seen in labrids and scarines. Although the possession of a secondarily modified PJA was correlated with increased diversification rates, this pattern is better explained by the evolution of extreme dichromatism (and other social and behavioral characters relating to sexual selection) within *Scarus *and *Chlorurus*. The PJA-innovation hypothesis also fails to explain the most dominant aspect of labrid lineage diversification, the radiation of the julidines. We suggest that pharyngeal jaws might have played a more important role in enabling morphological evolution of the feeding apparatus in labrids and scarines rather than in accelerating lineage diversification.

## Background

Labrid fishes comprise roughly 600 species and inhabit tropical and temperate marine habitats around the world. They are an ecologically dominant component of major reef systems [1] and display a staggering degree of trophic and morphological diversity [2-5]. Members exploit nearly all known feeding niches available to fishes including algae, fish, zooplankton, ectoparasites, crabs, polychaetes, mollusks, amphipods, and echinoderms [6], range in size from a few grams to over 100 kg, and exhibit high diversity in cranial and axial morphology [3,4,7,8]. Recently, the parrotfishes (subfamily Scarinae), which constitute one of the major groups of reef herbivores and bioeroders [9], have been recognized as a subclade of labrids [5,10]. One classic explanation for both the species richness and the ecomorphological diversity of labrids is that this clade has evolved a key innovation in the form of modified pharyngeal jaws that has fueled their subsequent radiation [11-14].

Percomorph fishes possess two sets of jaws: oral jaws which function in prey capture and manipulation, and pharyngeal jaws which usually aid in processing food and moving it to the esophagus. Like cichlids, which are also widely recognized for their exceptional functional diversity and species richness, labrids exhibit a highly modified condition of the pharyngeal jaw apparatus (PJA) referred to as pharyngognathy [12,14,15]. Pharyngognathy involves united left and right lower jaw elements (fifth ceratobranchials), a muscular sling connecting the neurocranium to the united fifth ceratobranchials, and a mobile diarthrotic articulation of the upper pharyngeal jaws with the neurocranium [12,14,15]. One of the most species-rich groups of labrids, the parrotfishes, exhibit further modifications of the PJA that are associated with forceful grinding [13,16,17]. These include a laterally expanded fourth epibranchial, laterally compressed upper pharyngeal jaws (pharyngobranchials), an anterior muscular sling through novel attachments of the transversalis ventralis muscle (complementing the existing posterior muscle sling), a well developed sliding joints between the pharyngobranchial, neurocranium and epibranchial that permit extensive anterior-posterior motion of the upper jaw, and a posterior to anterior progression of ordered tooth tows on the lower pharyngeal jaws. These modifications are thought to enable trophic diversification by allowing the pharyngeal jaws to take on enhanced functions in prey processing, freeing the oral jaws to become specialized for prey capture [15].

The labroid pharyngeal jaw condition has been proposed to be a key innovation [18] that underlies putative adaptive radiation in cichlids [15] and labrids [12-14]. Recent studies have examined the role of the pharyngeal jaws in shaping cichlid morphological diversification and cladogenesis [19-22]. However, the hypothesis that pharyngeal jaws have influenced labrid diversity has never been explicitly tested (though it recently received some support from Mabuchi and colleagues [11] who demonstrated that the PJA has evolved independently in labrids and a clade which includes cichlids, pomacentrids, and embiotocids). Similarly, although structural and functional innovations of the scarine pharyngeal jaw to allow the processing of algae and coral skeletons are thought to underlie the ecological radiation of this clade in reef and seagrass habitats [17,23], the influence of this trait upon parrotfish diversification patterns has never been studied.

In Schluter's framework of ecological adaptive radiation, key innovations are one mechanism that provides ecological opportunity [24]. These traits are hypothesized to trigger adaptive radiations by enabling a lineage that evolves the innovation to exploit a range of previously unavailable niches. The filling of niche space is expected to proceed rapidly. One of the central predictions of a key innovation hypothesis in this framework then is concordance between the acquisition of the key innovation and a shift in lineage diversification rate [25-27].

Here we evaluate this aspect of the key innovation hypothesis by testing whether the evolution of modified pharyngeal jaws has accelerated lineage diversification within labrid fishes. The current lack of knowledge of phylogenetic relationships among major percomorph groups prevents a sister group comparison between labrids and their outgroup. However labrids remain an especially good group to address this question because the nested radiation of parrotfishes within them provides the opportunity to examine both ancient and recent signatures of pharyngeal jaw innovation on patterns of diversification. We assembled the largest time-calibrated phylogeny of labrids to date and used comparative methods to assess the impact of the pharyngeal jaw modifications on diversification rate. We asked three general questions:

### 1. Did pharyngeal jaw innovation trigger rapid lineage diversification as part of an adaptive radiation?

If specialized pharyngeal jaws enabled labrids or parrotfishes to adaptively radiate along ecological axes, we would expect to see a pattern where diversification after the acquisition of the trait was rapid (as lineages exploited newly available niches) and then slowed through time (as this niche space became filled) [24,26,28]. We tested for this pattern, which has recently been identified as one of the ten key signatures of adaptive radiations [25], in two ways: first using the MCCR test of Pybus and Harvey [29] which tests for a slowing of diversification rates through time and second, by directly comparing the fit of density dependent models of cladogenesis to models where diversification is not a function of clade richness [28]. We applied these methods to both the entire timetree to test for this signature of adaptive radiation in the initial diversification of labrids, and within parrotfishes, to test for adaptive radiation following the evolution of pharyngeal jaws modified for grinding.

### 2. Are diversification rate shifts within labrids and scarids temporally concordant with the pharyngeal jaw innovations?

If PJA innovations have been primarily responsible for diversification within labrids and parrotfishes, we can make three further predictions. First, since key innovations are thought to trigger increased diversification, we would expect the overall rate of labrid diversification to be high compared to other percomorph fishes. Second, if the PJA is the main cause of labrid species richness, any additional diversification rate increases should be restricted to relatively small subclades. If a large fraction of labrid richness occurs in young, fast-evolving subclades temporally removed from the labrid root, then the PJA is a weak explanation for standing labrid diversity even if the PJA played some role in initially establishing the clade in diverse environments. Third, if the pharyngeal mill in parrotfishes were a key innovation that triggered a further adaptive radiation, we would expect to see diversification rates increase at or near the origin of this clade.

### 3. Does the character state of the pharyngeal jaws predict diversification rate?

Key innovation hypotheses predict that lineages with the innovation should diversify more quickly than lineages that lack the trait [30]. We used BiSSE [31], a recently developed comparative method, to test whether labrid lineages with a modified pharyngeal mill (i.e. the parrotfishes) have diversified more quickly than those with the labroid PJA. The sister group of labrids is currently not known [5,11,32]. This lack of phylogenetic resolution prevented us from testing whether the labroid PJA itself was associated with faster rates of diversification than the generalized percomorph condition.

## Results

### Divergence time analysis

**Figure 1:**
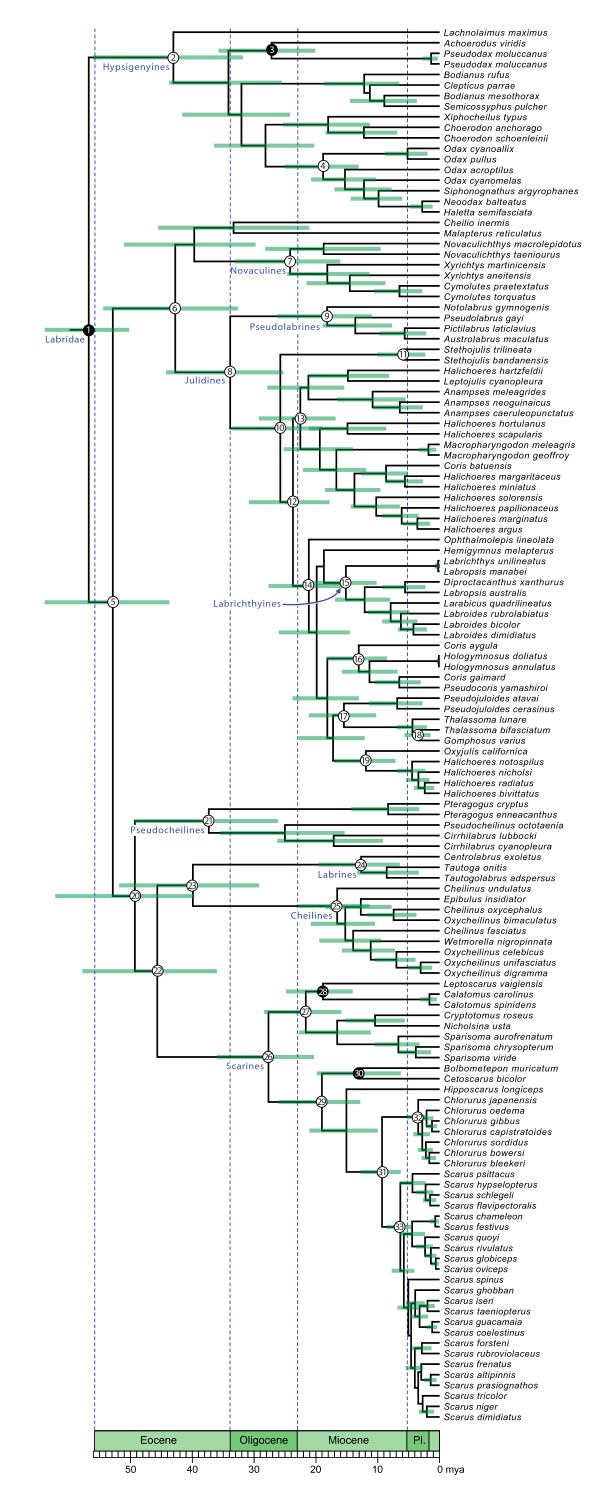
**Time-calibrated phylogeny (chronogram) of labrid fishes based on mitochondrial and nuclear sequences**. Filled circles indicate fossil-calibrated nodes (Table 7). Bars indicate 95% HPD for divergence time estimates. Focal nodes indicated by circles (Table 1). Posterior probabilities for all focal nodes was 90%. Scale bar at the bottom is in million of years since the present.

Our BEAST analysis produced a well-resolved phylogeny of 131 labrid species that was in good agreement with previous work (Fig. 1) [5,33]. A recent divergence time study of labrids treated the crown age as fixed at 55 MY [33]. Our analysis recovered an almost identical crown age of labrids even though we assigned far more liberal constraints to this node (50-120 MY) (Fig. 1; Table 1). Our estimate of the split between scarines and cheilines + labrines (46 MY, 95% HPD:36-58) is consistent with both the CR (53 MY) and PL (36 MY) estimates of Smith et al [33]. In contrast, our estimates for the age of crown scarines (28 MY, 95% HPD:20-36) excludes both Streelman et al.'s [23] age of 42 MY and Smith et al.'s [33] estimate of 17 MY. The rest of our estimates within parrotfishes including the age of the seagrass clade, reef clade, *Scarus *+ *Chlorurus *and crown ages of those genera are slightly older than those Smith et al., [33] though in almost all cases their mean is captured in our 95% credible interval.

**Table 1:** Ages of focal nodes in Fig. 1.

node	description	mean age (MY)	95% HPD (MY)
**1**	**crown labrids**	**57**	**50-69**
2	hypsigenyines	43	32-56
**3**	***Achoerodus *vs *Pseudodax***	**27**	**20-36**
4	odacids	19	13-25
5	non-hypsigenyines	53	44-66
6	julidines + novaculines	43	33-54
7	novaculines	24	16-33
8	julidines	34	25-44
9	pseudolabrines	18	11-26
10	*Stethojulis *vs. *IP Halichoeres*	26	19-33
11	*Stethojulis*	6	2-10
12	fast-evolving julidines from MEDUSA analysis	24	18-31
13	IP *Halichoeres *et al.	22	17-29
14	labrichthyines vs. *Ophthalmolepis*	21	15-28
15	labrichthyines	15	10-21
16	*Coris *+ *Pseudocoris *+ *Hologymnosus*	13	9-18
17	*Pseudojuloides *vs. *Thalassoma*	15	10-21
18	*Thalassoma *+ *Gomphosus*	4	2-6
19	New World *Halichoeres *et al.	12	7-17
20	pseudocheilines vs. labrines, cheilines, and scarines	49	40-62
21	pseudocheilines	37	26-49
22	labrines + cheilines + scarines	46	36-58
23	labrines + cheilines	40	29-52
24	labrines	13	6-20
25	cheilines	17	11-23
26	scarines	28	20-36
27	seagrass parrotfishes	22	16-28
**28**	***Calotomus + Leptoscarus***	**19**	**14-25**
29	reef parrotfishes	19	13-26
**30**	***Bolbometopon *vs. *Cetoscarus***	**13**	**6-10**
31	*Scarus + Chlorurus*	9	6-13
32	*Chlorurus*	4	2-5
33	*Scarus*	6	4-9

Nodes in bold were fossil-calibrated (Table 7).

### Diversification analysis

**Figure 2:**
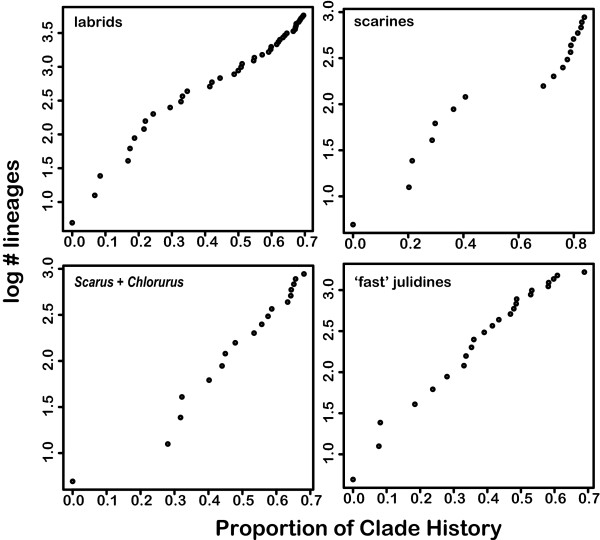
**Lineage through time plots of early history of labrids, scarids, and two subclades identified by MEDUSA analysis as diversifying exceptionally rapidly (Fig. 3)**. Proportion of clade history is measured from the root node of each clade.

A lineage through time plot revealed that the log number of lineages appeared to accumulate at a roughly constant rate in the early history of labrids. This pattern is expected for lineages where diversification rate has been constant [34], suggesting that diversification in early labrid history was not initially fast (Fig. 2). This interpretation was supported by the MCCR test for labrids which failed to reject the hypothesis of a constant diversification rate (Table 2). Although the exponential density dependent model fit the labrid data best, the 95% credible set of models based on the calculation of Akaike weights [35] did not exclude the pure-birth model. A lineage through time plot for scarines revealed fewer lineages in their early history than expected under a constant rates model. This result was reinforced by a nonsignificant MCCR test (Table 2), suggesting that explosive scarine diversification did not accompany the evolution of modified pharyngeal jaws. An exponential model of density-dependent diversification fit the scarines best but the 95% credible interval did not exclude the pure birth model (Table 3).

**Table 2:** MCCR results for tests of labrid subclades.

clade	richness	sampled	gamma	p
labrids	573	131	-0.48	>0.99
scarines	96	42	0.95	0.96

**Table 3:** Fit of diversification models from Rabosky and Lovette[27] to the first 70% of labrid clade history (first 85% of scarine history).

clade	Akaike Weight				lnL best model
		
	PB	DDX	DDL	LD	
labrids	0.32	**0.36**	0.32	0.00	-29.98
scarines	0.32	**0.36**	0.32	0.00	-18.59
*Scarus *+ *Chlorurus*	0.33	**0.34**	0.33	0.00	4.82
fast-evolving julidines	0.01	0.14	**0.84**	0.01	-15.44

Akaike weights [35] indicate the relative strength of support (ranging from 0 to 1) for four candidate models: PB = pure birth, DDX = density dependent (exponential), DDL = density dependent (logarithmic), LD = linear decline. Best model indicated in bold.

MEDUSA (Modeling Evolutionary Diversification Using Stepwise AIC), a recently developed comparative method that integrates taxonomic and phylogenetic information, allows exceptionally radiating clades to be identified on an incompletely sampled phylogeny [36,37]. MEDUSA analysis revealed that the background rate of labrid diversification, (0.086 lineages/MY) is somewhat higher than the average rate of diversification of percomorph (0.081 lineages/MY) and ostariophysan (0.067 lineages/MY) fishes as well as most major tetrapod lineages [36,37] (Table 4). We found evidence for two significant rate shifts within labrids, though neither of these corresponded to the predictions generated by the PJA key-innovation hypothesis (above). The first corresponded to the origin of a clade comprising *Scarus *and *Chlorurus *(S-C clade hereafter). The net diversification rate of the stem S-C lineage is only modestly higher than the background rate of labrid diversification due to a long branch leading from the split with *Hipposcarus *to the crown group (Table 4). However rates within the crown S-C clade (r = 0.23, ε = 0.76) were approximately 2.5 times greater than the net diversification rate of other labrids. The second rate shift occurred on the branch leading to most of the julidine diversity including the Indo-Pacific *Halichoeres*, the New World *Halichoeres*, and the Labrichthyinae. The rate within this lineage was approximately twice that of labrids diversifying at the background rate and roughly equal to the rate of diversification within the crown S-C clade (Table 4). We fit diversification models to both of the fast-evolving clades identified by MEDUSA analysis to explore whether their patterns were consistent with adaptive radiation. The scarines did not strongly favor a density dependent model (Table 3), suggesting that their early diversification was not explosive. This result was reinforced by a convex lineage through time plot, indicating fewer than expected species in their early history. In contrast, the fast evolving julidines showed some evidence for adaptive radiation both by model fitting which strongly favored density dependent models (Table 3), and a concave lineage through time plot (Fig. 2) indicating more species than expected in their early history.

**Table 4:** The tempo of labrid diversification.

# of shifts	clade	r	ε	AIC	ΔAIC
0 (whole-tree birth-death model)	whole tree	0.053	0.912	307.76	
1	1 (*Scarus *+ *Chlorurus*)	0.100	0.954	301.45	6.41
2	2 ('fast' julidines)	0.199	0.000	284.21	17.24
--	background	0.086	0.000	--	--

Clade number refers to rate shifts identified in Fig. 2. r is the net diversification rate, ε is the extinction fraction (d/b). AIC and ΔAIC show improvement of AIC score over a constant rates birth-death model as clades are allowed to change rates. Background shows background rates of other labrid clades under the two-rate model.

We tested for a correlation between diversification rate and the presence of a parrotfish pharyngeal mill using BiSSE [31]. Our results found strong support in favor of a model where pharyngeal mill-equipped lineages diversified ~4X faster than lineages with the typical labrid pharyngeal jaw apparatus (Table 5). However we were concerned with the possibility of a trickle-down effect from the S-C clade given that our MEDUSA analysis found a rate increase for this group. To investigate this further, we reran BiSSE with the S-C clade excluded and found no support for a two-rate model (Table 5). Furthermore, the speciation estimates in this unsupported two-rate model were nearly identical (0.057 with pharyngeal mill vs. 0.053 without) suggesting that diversification rates in parrotfish genera besides *Scarus *and *Chlorurus *are similar to rates in other labrids.

**Table 5:** BiSSE negative log-likelihoods of constrained (λ_0 _= λ_1_) and unconstrained models for the modified pharyngeal jaw character (found in Scaridae).

Character	-LnL (Unconstrained)	-LnL (Constrained)	ΔLnL	λ0	λ1
PJM (Scarines)	**474.35**	482.71	8.36 (P << 0.01)	0.059	0.234
PJM (No *Scarus-Chlorurus*)	388.54	**388.48**	0.06 (P > 0.05)	0.057	0.053
Extreme Dichromatism	**466.28**	482.32	16.004 (P << 0.01)	0.056	0.281

Parameter estimates of the constrained (λ_0 _= λ_1_) and unconstrained models for the pharyngeal jaw modifications found in all Scaridae (=state 1).

The S-C clade comprises the most sexually dichromatic, haremic, and territorial of parrotfishes [23] and it has been suggested that sexual selection has played a dominant role in their diversification [23,38]. To test whether extreme sexual dichromatism was a better explanation of diversification rates than a pharyngeal mill, we performed another BiSSE analysis with the parrotfishes in the S-C clade coded as 1 and the rest of the phylogeny as state 0. A comparison of likelihood scores favored the extreme dichromatic model over the pharyngeal mill (Table 5).

## Discussion

### Did the evolution of the labrid pharyngeal jaw trigger rapid lineage diversification?

A common element of many key innovation hypotheses is that the trait is linked to rapid diversification [18] and adaptive radiation [24]. Although we found the Labridae as a whole to have diversified rapidly compared to other percomorphs, we also found no evidence for a pulse of cladogenesis coincident with the origin of the clade followed by declining rates as predicted by models of adaptive radiation [28,29]. Instead, we found that cladogenesis in the early history of labrids proceeded in a fairly log-linear manner consistent with a model where net diversification rates were constant. Thus, we find no direct support for the hypothesis that the PJA triggered rapid lineage diversification as part of an adaptive radiation (*sensu *Schluter [24]). High rates of extinction have been shown to erase the signature of adaptive radiation [26,28,39] and so one possibility is that background extinction within labrids has masked PJA-facilitated diversification. However other marine fish clades of roughly similar ages with less trophic diversity and species richness do retain the signature of exceptionally rapid initial diversification [40]. Thus we are skeptical that exceptionally high extinction rates have masked the signal of explosive lineage diversification in labrids. Further evaluation of extinction rates across labrid history is hampered by their poor fossil record. In any case, our analyses cast doubt on the PJA-key innovation as a strong general explanation of labrid species richness because over 40% of non-scarine labrid diversity can be attributed to the julidine rate shift, an event which occurred ~30 MY after the origin of the labrid PJA.

Our findings are similar to those from recent studies of cichlid diversity. Although the pharyngeal jaw has been suggested to underpin species richness in this family as well [15], recent phylogenetic analyses have found that the major diversification rate shift which lead to the origin of most of the diversity of East African haplochromines (~1800 species) occurred within the last 2.4 MY, well after the evolution of the PJA [20-22]. Instead, diversification patterns appear to be strongly correlated with the evolution of specific behavioral and sexual characters [38] such as mouth brooding and egg spots on the anal fins [20].

### Is the parrotfish pharyngeal mill a key innovation that explains scarine biodiversity?

We similarly found weak evidence in favor of the pharyngeal mill key innovation hypothesis. The MCCR test did not support the hypothesis that early parrotfish diversification had slowed through time and fitting of diversification models did not favor density dependent models (Tables 2, 3). Once again it is possible that high extinction rates have masked this signature although we regard this as less likely since scarines are considerably younger than crown labrids. Although we did find a significant increase in the rate of scarine diversification relative to other labrids, this rate increase was restricted to a clade comprising two very young genera of parrotfishes rather than at or near the root of the entire clade. The strongest evidence supporting the idea that a pharyngeal mill has contributed to scarine biodiversity comes from our BiSSE analysis which found a high correlation between the possession of a pharyngeal mill and the diversification rate. However we suggest that this result is driven by trickledown effects of the rate increase on *Scarus *+ *Chlorurus*. This was supported by our BiSSE reanalysis which showed that the diversification rate in other scarines was very similar to the average labrid diversification rate. We suggest that the most likely cause of diversification in the S-C clade is the evolution of extreme male breeding coloration and reproductive behaviors through sexual selection [23]. Other contributing biogeographic factors are considered in Smith et al., [33] and include Pliocene/Pleistocene fluctuations in sea level and the closing of the Isthmus of Panama.

### What explains the rate shifts in julidines?

The julidines have been recognized as one of the largest of all coral reef fish radiations [5]. For the first time we show that this radiation was exceptionally fast, with a net diversification rate of 0.19 species/MY. Recently Alfaro et al. [41] found evidence of rapid diversification of reef-associated tetraodontiform families during the late Oligocene and early Miocene. The mean age estimate of the julidine rate increase (~24 MY) falls at the end of the Oligocene, suggesting that similar factors may underlie the diversification of julidines and possibly other major reef-associated fish clades. These include the closing of the Tethys and the collision of the Australia New Guinea plate with SE Eurasia [41-44].

### Do trophic key innovations drive species diversification?

The key innovation hypothesis has been invoked to explain both the species richness and phenotypic diversity of labrids [11,15,18,24,45]. Although these two aspects of a radiating clade are often conflated, it is important to point out that diversification and phenotypic evolution need not be strongly linked [46,47]. Our results show that pharyngeal jaw innovations provide weak explanations for the major patterns of species richness observed at relevant levels of labrid and scarine phylogeny. However it is currently not known if the labrid PJA or the pharyngeal mill of parrotfishes could have acted as a key innovation to spur rates of functional evolution as it has in cichlids [19]. The wealth of studies on labrid functional evolution suggests that this might be true. Multiple studies have established that labrids are functionally [3,48] and trophically diverse [5,49,50], that their functional diversity is partitioned unevenly across extant clades [3], and that they display complex patterns of functional evolution over their history [5]. Similarly, it is possible that the parrotfish pharyngeal mill is associated with a greater than expected amount of functional and morphological diversity observed in scarines relative to other labrid clades [3]. Increasingly sophisticated methods exist for answering questions about patterns and rates of morphological evolution [51-53] but have yet to be applied to test key innovation hypotheses.

Our results add to a growing body of work on diversification patterns in fish clades with modified pharyngeal jaws [20-22]. Together these studies cast doubt on the hypothesis that the pharyngeal jaw innovation itself is directly responsible for observed patterns of species richness in fishes. It is possible that pharyngeal jaw innovations influence diversification rates by allowing clades to establish ecological 'footholds' in novel environments [18] or in ways that are context-dependent [54]. However, these formulations of key innovation hypotheses are difficult to test with the suite of comparative methods currently available to evolutionary biologists [55]. In contrast, predictions about the influence of pharyngeal jaw modifications on evolution of other trophic characters are more direct and lend themselves to hypothesis testing [19]. We suggest that pharyngeal jaw innovations do not constitute a general explanation for patterns of labrid or scarine diversity but that the hypothesis that this trait represents a key innovation might still be useful in explaining patterns of morphological and functional evolution within these clades.

## Conclusion

Labrids diversified rapidly relative to other percomorphs. However there is no evidence that pharyngeal jaw innovations triggered explosive lineage diversification within either labrids or scarines. Even if pharyngeal jaw evolution triggered adaptive radiation with accelerated cladogenesis, over half of labrid richness can be attributed to two more recent diversification events where key innovations are not suspected as causes: one within the julidines and one within the most dichromatic of parrotfishes, *Scarus *and *Chlorurus*. The similarity of these results to similar studies of diversification patterns in cichlids suggests that the pharyngeal jaws-as-key-innovations hypothesis should not be invoked as a general explanation for the species diversity in either family though it may have utility in explaining patterns of ecomorphological diversity.

## Methods

### Divergence time estimation

We downloaded GenBank sequence data for 131 labrid species and 17 outgroups from three previously published studies: Westneat and Alfaro [5], Clements et al. [10], and Smith et al. [33] for two mitochondrial (12S, 16S) and two nuclear (tmo4c4, RAG2) genes. Genbank accession numbers are given in Additional FIle 1.

We aligned the mitochondrial gene sequences to previously published models of secondary structure in a text editor and used the Clustal [56] module of Geneious [57] to align the protein coding nuclear genes and concatenate the matrix. We compared three possible partitioning schemes of the concatenated data using Bayes factors based on the marginal likelihood: all genes together (one partition), separate partition for each gene (four partitions), and separate partitions for 12S and 16S plus codon positions within genes (eight partitions). We assigned each partition a GTR + I + G model. In addition, we examined an eight partition scheme with an HKY + G model to assess whether a more simple substitution model better fit the data. After comparing Bayes factors in Tracer [58] (Table 6) we used the best of the four partitioning schemes (the eight parameter GTR + I + G model) to estimate divergence times using BEAST 1.4 [59]. However, we found that all four models produced nearly identical results where the ages of focal nodes differed by less than +/- 1 MY. We constrained four clades in the analysis on the basis of the labrid fossil record (Table 7). In each case the age of the fossil served as a hard bound on the minimum age of the constrained clade. To mitigate against the effects of truncated prior distribution [60,61] we assigned exponential priors to the constrained nodes where the 95% upper limit on the prior reflected our best guess for the maximum age of the clade based on the fossil record.

**Table 6:** Marginal likelihood and Bayes factor comparisons for partitioning strategies explored for divergence time analysis.

Partition	Substitution Model	Marginal lnL	BF 1P	BF 4P	BF 8P (HKY + G)	BF 8P (GTR + I +G)
concatenated (1P)	GTR + I G	-47075.5	--	114.3	186.8	368.1
by gene (4P)	GTR + I G	-46812.3	-114.3	--	72.0	253.8
by gene codons + by mit. gene (8P)	HKY + G	-46646.5	-186.3	-72.0	--	181.8
by gene codons + by mit. gene (8P)	GTR + I G	-46228	-368.1	-253.8	-181.8	--

BF is log10 Bayes factor comparison in support of each model and partitioning scheme.

**Table 7:** Bounds on fossil calibrated nodes.

calibration	description	min/95%	mean
1	crown labridae	50/120	83.5
2	crown hypsigenyines (except *Lachnolaimus*)	20/50	30
3	crown seagrass parrotfishes	14/50	26
4	split *Bolbometepon *vs. *Cetoscarus*	5.3/50	23.5

Min/95% refers to the minimum age of the fossil and the age at the 95% exponential distribution. The mean describes the shape of the exponential distribution.

#### Crown Labridae

(Fig. 1, node 1): The fossil *Phyllopharyngodon longipinnis *from the Middle Eocene of the Monte Bolca (50 MY) [62] is the earliest known labrid and is considered to be a stem hypsigenyine, providing a minimum estimate for the age of crown labrids. We placed an upper bound for the age of the crown labrids at 120 MY to reflect our belief that it is unlikely that labrids are much older than the oldest known acanthomorph fossils, dated 90-110 MY [63,64].

#### Crown hypsigenyines (except *Lachnolaimus*)

Trigonodon (Fig. 1, node 3): The fossil *Trigonodon jugleri *[17,65], known from the Early Miocene (20 MY), is a stem chiseltooth wrasse (genus *Pseudodax*). In a recent molecular phylogeny of labrids, Westneat and Alfaro [5] recover the clade *Pseudodax *+ *Achoerodus *as the sister to all other hysigenyines except for *Lachnolaimus*. On the basis of this placement, we constrained the crown age of hypsigenyines (excluding *Lachnolaimus*) to be 20 MY. We assigned an upper limit of 50 MY to reflect our belief that this split is likely to be younger than the first appearance of stem hypsigenyines (above).

#### Crown seagrass parrotfishes

(Fig. 1, node 27): A fossil parrotfish, *Calotomus preisli *[65] is known from the Middle Miocene (14 MY). Recent molecular studies place *Calotomus *within the 'seagrass' [23] clade of parrotfishes though there is some ambiguity about the exact position of the lineage within this clade [23,33]. We calibrated the minimum age of the 'seagrass' parrotfishes using this fossil and assigned a maximum age of 50 MY to reflect our belief that this split is younger than the age of the oldest known fossil labrids (above).

#### Split *Bolbometopon *vs. *Cetoscarus*

(Fig. 1 node 29) Fossil elements belonging to the genus *Bolbometopon *are known from the late Miocene (5.3 MY) [17,65] and we used this as a minimum age of the split between *Bolbometopon *and *Cetoscarus*. We assigned a maximum age of 50 MY to this split to reflect our prior belief that *Bolbometopon *and *Cetoscarus *diverged before the age of the earliest known labrids (above).

We ran the BEAST MCMC sampler for 50 million generations sampling every 1000 generations. We assessed convergence visually using Tracer [58] to plot of likelihood versus generation and estimate the effective sample size (ESS) of all parameters. As an additional check that the sampler converged on the target distribution, we repeated the analysis with separate starting trees five times.

### Diversification Analysis

**Figure 3:**
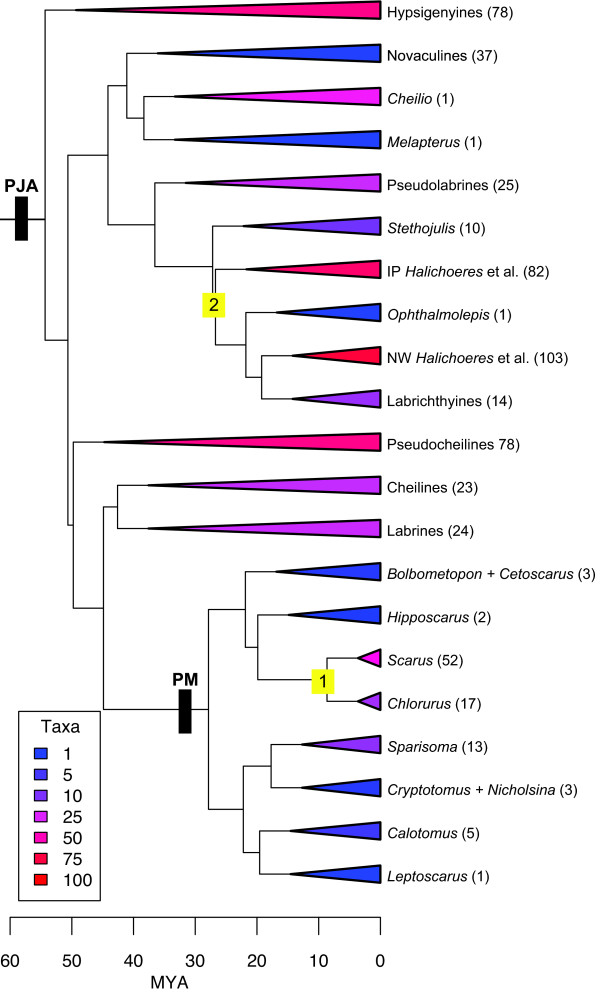
**Phylogenetic placement of diversification rate shifts and pharyngeal jaw modifications**. Tip clade richness follows names with warmer colored tip triangles indicate subclades with greater species richness. Numbered branches indicate position of two diversification rate increases. Origin of labroid pharyngeal jaw apparatus (PJM) and parrotfish pharyngeal mill (PM) indicated by black rectangles. Tree backbone is taken from Figure 1. Species richness and taxonomic membership of major subclades given in Table 8.

We used the LASER package [66] in R to generate lineage through time plots for labrids, scarines and the two fast-evolving subclades identified by MEDUSA (Fig. 3). We tested whether rapid lineage diversification characterized the origin of labrids, and parrotfishes using the MCCR test (Pybus and Harvey, 2001) which compares the distribution of branching events on the observed tree to that expected under a pattern of constant diversification. To account for incomplete taxon sampling we constructed a null distribution of the test statistic (gamma) with 1000 replicates that reflected the subsampling of the clade in question [29]. For example, we simulated the evolution of 1000 573-taxon trees (to reflect current estimates of labrid diversity) and pruned them to 131 tips (to reflect our sampling) using the mccrTest in the Laser package for R. Total and sampled richness for each of these groups is reported in Table 8.

**Table 8:** Total and sampled richness for MEDUSA analysis.

taxon	richness	included genera
Bolbometopon + Cetoscarus	3	Bolbometopon, Cetoscarus
Calotomus	5	Calotomus
Cheilines	23	Epibulus, Oxycheilinus, Cheilinus, Wetmorella
Chlorurus	17	Chlorurus
Cryptotomus+Nicholsina	3	Cryptotomus, Nicholsina
Hipposcarus	2	Hipposcarus
Hypsigenyines	78	Lachnolaimus, Achoerodus, Pseudodax, Bodianus, Semicossyphus, Clepticus, Neoodax, Odax, Xiphocheilus, Choerodon
IP Halichoeres	82	Leptojulis, Halichoeres, Anampses, Macropharyngodon, Coris batuensis
Labrichthyines	14	Labrichthys, Diproctacanthus, Labropsis, Larabicus, Labroides
Labrines	24	Labrus, Lappanella, Ctenolabrus, Acantholabrus, Tautogalabrus, Tautoga, Symphodus, Centrolabrus
Leptoscarus	1	Leptoscarus
Novaculines	37	Novaculichthys, Novaculoides, Xyrichtys, Iniistius, Cymolutes
NW Halichoeres et al.	103	Hemigymnus, Coris, Pseudocoris, Hologymnosus, Thalassoma, Gomphosus
Ophthalmolepis	1	
Pseudocheilines	78	Cirrhilabrus, Pseudocheilinus, Pteragogus
Pseudolabrines	25	Austrolabrus, Pictilabrus, Notolabrus, Pseudolabrus
Scarus	52	Scarus
Sparisoma	13	Sparisoma
Stehojulis	10	Sparisoma
Cheilio	1	Cheilio
Melapterus	1	Melapterus

Species richness from Fishbase [70].

We restricted our lineage through time plots and fitting of diversification models to the early history of focal clades for two reasons. First, our questions about the relationship between the acquisition of pharyngeal jaw characters and lineage diversification predict that adaptive radiation would leave a signature on the early evolutionary history of labrid groups. Second, the previous phylogenetic studies which provide the sequence data for our analyses were designed to capture the splitting events among major lineages but not to resolve species-level relationships within diverse genera [4,23,33]. Thus we expect our phylogeny to capture early splitting events among tribes and genera and to undersample more tipward splits. Incomplete sampling of more recent splits may cause an apparent decline in net diversification towards the present, creating the potential for artifactual rejection of a constant-rates model. To avoid this problem, we followed the approach of Nee et al. [67] and fit diversification models (and restricted lineage through time plots) to the first 70% of the timetree (from the root) of labrids, fast-evolving julidines, and *Scarus *+ *Chlorurus*. We included the first 85% of the scarine timetree because the taxonomy and phylogeny of this clade has been long studied [17,23,33,68] and we are confident that the only unsampled splitting events in our tree are within the young clades *Scarus*, *Chlorurus*, and *Sparisoma*. Four models of diversification were fit to the labrid and scarine timetrees and the two fast-evolving clades identified by MEDUSA using maximum likelihood: a constant-rates pure birth; logistic and exponential density-dependent; and linear decline in which net diversification decreased through time at a rate that is independent of clade size [27]. We modified R code kindly provided by Dan Rabosky to fit these four models of diversification to the time-truncated phylogeny. For each model, the difference between its AIC score (AIC) and that of the best-fitting model was calculated as well as the Akaike weight. All model-fitting analyses were done in R [69].

To identify periods of exceptional diversification in the history of labrids we used MEDUSA (Modeling Evolutionary Diversification Using Stepwise AIC) a recently developed comparative method that combines phylogenetic and taxonomic information to estimate rate shifts on a phylogeny [36]. We first compiled taxonomic species richness data from FishBase [70] for the major clades of the timetree. Then we pruned the tree down so that each of these clades was represented by a single tip species. In pruning the tree we strived to preserve the maximum amount of phylogenetic resolution possible that would still allow the entirety of labrid species richness to be distributed among the tips. Thus we retained a single representative of the genus *Scarus *in the pruned tree and assigned it the richness of the genus (52 species) because we could not confidently divide the richness further among the tips we sampled. Assignment of unsampled species richness was based upon the membership and placement of labrid tribes and subclades from Fig. 1 and previous taxonomic and phylogenetic studies [5,10,17,23,43,71,72].

MEDUSA involves the stepwise addition of rate shifts on the pruned topology. In the first iteration, the AIC score of a birth-death model across the diversity tree was compared to a model where both rates were allowed to shift on the optimal branch (in this case, the branch leading to *Scarus *+ *Chlorurus*). If the rate shift substantially improved the AIC score, we retained the shift and repeated the procedure, comparing the two rate tree to a tree where the rate was allowed to optimally shift on a third branch. We repeated this procedure until the addition of parameters resulted in AIC improvements of less than 4 units (indicating moderate support of the data for the model in an AIC framework [35]). Code to perform MEDUSA analysis is distributed in the Geiger package [73] for R [69].

We used BiSSE [31], implemented in Mesquite [74] to test key innovation hypotheses to explain patterns of diversification in labrid fishes. BiSSE [31] provides a likelihood-based test of whether a discrete character (in this case the presence or absence of a modified pharyngeal mill) influences the rate of lineage diversification. First we tested whether the evolution of modified grinding pharyngeal jaws facilitated rapid diversification in parrotfishes relative to other labrids by coding the tips in Fig. 1 for presence/absence of a pharyngeal mill. Second, we repeated the first analysis excluding the extremely dichromatic genera *Scarus *and *Chlorurus *to test whether rapid diversification within this clade was driving significance across all scarines. Finally we tested whether the evolution of extreme dichromatic coloration in *Scarus *and *Chlorurus *was a better explanation of diversification rate than the acquisition of a pharyngeal mill. In all cases, BiSSE was used to compute likelihoods of our empirical data (timetree and character states at the tips) under two models, a constrained and unconstrained model. The unconstrained model had all parameters (i.e. λ, μ, q) free to vary while the constrained model forced the speciation rates for both character states to be equal (λ_0 _= λ_1_). Two times the difference in log-likelihoods was computed and a χ^2^-distribution with a single degree-of-freedom was used to test for significance.

## Authors' contributions

MEA, CDB, and BB designed study. MEA, CDB, BB, and PCW performed analyses. MEA, CDB, and BB wrote the manuscript. All authors read and approved the final manuscript.

## Supplementary Material

Additional file 1 **Genbank accession numbers for sequences used in this study**. Genbank accession numbers for sequences used to create data matrices for phylogenetic analysis. Click here for file
