# Supplementary material for: Does evolutionary innovation in pharyngeal jaws lead to rapid lineage diversification in labrid fishes?
Source: BMC Evol Biol. 2009 Oct 22;9:255. doi: 10.1186/1471-2148-9-255 (PMC2779191; doi:10.1186/1471-2148-9-255)
Supplement: Additional file 1 — Genbank accession numbers for sequences used in this study. Genbank accession numbers for sequences used to create data matrices for phylogenetic analysis. [file 1471-2148-9-255-S1.DOC]

Additional File 1: Genbank Accession Numbers for sequences used in this study.

| Genus | Species | 12S | 16S | Tmo 4c4 | RAG2 |
| --- | --- | --- | --- | --- | --- |
| *Abudefduf* | *saxatilis* | AY279570 | AY279673 | AY279776 | AY279879 |
| *Achoerodus* | *viridis* | AY279574 | AY279676 | AY279780 | AY279883 |
| *Amblyglyphidodon* | *leucogaster* | AY279568 | AY279671 | AY279774 | AY279877 |
| *Amphistichus* | *argenteus* | AY279571 | AY279674 | AY279777 | AY279880 |
| *Anampses* | *caeruleopuntatus* | AY279575 | AY279677 | AY279781 | AY279884 |
| *Anampses* | *meleagrides* | DQ164139 | DQ164174 | -- | -- |
| *Anampses* | *neoguinaicus* | AY279576 | AY279678 | AY279782 | AY279885 |
| *Austrolabrus* | *maculatus* | AY279577 | AY279679 | AY279783 | AY279886 |
| *Bodianus* | *mesothorax* | AY279578 | AY279680 | AY279784 | AY279887 |
| *Bodianus* | *rufus* | AY279579 | AY279681 | AY279785 | AY279888 |
| *Bolbometopon* | *muricatum* | EU601178 | AY081091 | EU601263 | EU601307 |
| *Calotomus* | *carolinus* | EU601179 | AY081092 | EU601264 | EU601308 |
| *Calotomus* | *spinidens* | EU601180 | EU601228 | EU601265 | EU601309 |
| *Centrolabrus* | *exoletus* | EU601224 | EU601260 | EU601303 | EU601353 |
| *Cetoscarus* | *bicolor* | EU601181 | EU601229 | EU601266 | EU601310 |
| *Cheilinus* | *fasciatus* | AY279580 | AY279682 | AY279786 | AY279889 |
| *Cheilinus* | *oxycephalus* | AY279581 | AY279683 | AY279787 | AY279890 |
| *Cheilinus* | *undulatus* | AY279582 | AY279684 | AY279788 | AY279891 |
| *Cheilio* | *inermis* | AY279583 | AY279685 | AY279789 | AY279892 |
| *Chlorurus* | *bleekeri* | EU601182 | EU601230 | EU601267 | EU601311 |
| *Chlorurus* | *bowseri* | EU601183 | EU601231 | EU601268 | EU601312 |
| *Chlorurus* | *capistratoides* | EU601184 | EU601232 | EU601269 | EU601313 |
| *Chlorurus* | *japanensis* | EU601209 | EU601250 | EU601288 | EU601338 |
| *Chlorurus* | *oedema* | EU601186 | EU601234 | AY081107 | EU601315 |
| *Chlorurus* | *sordidus* | AY279584 | AY279686 | AY279790 | AY279893 |
| *Choerodon* | *anchorago* | AY279585 | AY279687 | AY279791 | AY279894 |
| *Choerodon* | *schoenleinii* | AY279586 | AY279688 | AY279792 | AY279895 |
| *Cirrhilabrus* | *lubbocki* | AY279587 | AY279689 | AY279793 | AY279896 |
| *Clepticus* | *parrae* | AY279588 | AY279690 | AY279794 | AY279897 |
| *Coris* | *aygula* | AY279589 | AY279691 | AY279795 | AY279898 |
| *Coris* | *batuensis* | AY279591 | AY279693 | AY279797 | AY279900 |
| *Coris* | *gaimard* | AY279590 | AY279692 | AY279796 | AY279899 |
| *Cryptotomus* | *roseus* | AY279592 | AY279694 | AY279798 | AY279901 |
| *Cymolutes* | *praetextatus* | AY279593 | AY279695 | AY279799 | AY279902 |
| *Cymolutes* | *torquatus* | AY279594 | AY279696 | AY279800 | AY279903 |
| *Damalichthys* | *vacca* | AY279572 | AY279675* | AY279778 | AY279881 |
| *Dascyllus* | *trimaculatus* | AY279569 | AY279672 | AY279775 | AY279878 |
| *Diagramma* | *pictum* | AY279563 | AY279666 | AY279769 | AY279872 |
| *Diproctacanthus* | *xanthurus* | AY279595 | AY279697 | AY279801 | AY279904 |
| *Dischistodus* | *chrysopoecilus* | AY279567 | AY279670 | AY279773 | AY279876 |
| *Embiotoca* | *jacksoni* | AY279573 | AY279675 | AY279779 | AY279882 |
| *Epibulus* | *insidiator* | AY279596 | AY279698 | AY279802 | AY279905 |
| *Etroplus* | *maculatus* | AY279565 | AY279668 | AY279771 | AY279874 |
| *Gerres* | *cinereus* | EF095573 | EF095601 | -- | -- |
| *Gomphosus* | *varius* | AY279597 | AY279699 | AY279803 | AY279906 |
| *Haletta* | *semifasciata* | AY279656 | AY279759 | AY279862 | AY279965 |
| *Halichoeres* | *argus* | AY279598 | AY279700 | AY279804 | AY279907 |
| *Halichoeres* | *bivittatus* | AY279599 | AY279701 | AY279805 | AY279908 |
| *Halichoeres* | *hartzfeldi* | AY279600 | AY279702 | AY279806 | AY279909 |
| *Halichoeres* | *hortulanus* | AY279601 | AY279703 | AY279807 | AY279910 |
| *Halichoeres* | *margaritaceous* | AY279602 | AY279704 | AY279808 | AY279911 |
| *Halichoeres* | *marginatus* | AY279603 | AY279705 | AY279809 | AY279912 |
| *Halichoeres* | *miniatus* | AY279604 | AY279706 | AY279810 | AY279913 |
| *Halichoeres* | *nicholsi* | AY279605 | AY279707 | AY279811 | AY279914 |
| *Halichoeres* | *notospilus* | AY279606 | AY279708 | AY279812 | AY279915 |
| *Halichoeres* | *papilionaceus* | AY279609 | AY279711 | AY279815 | AY279918 |
| *Halichoeres* | *radiatus* | AY279607 | AY279709 | AY279813 | AY279916 |
| *Halichoeres* | *scapularis* | AY279608 | AY279710 | AY279814 | AY279917 |
| *Halichoeres* | *solorensis* | AY279610 | AY279712 | AY279816 | AY279919 |
| *Hemigymnus* | *melapterus* | AY279611 | AY279713 | AY279817 | AY279920 |
| *Hipposcarus* | *longiceps* | AY081075 | AY081093 | AY081110 | EU601318 |
| *Hologymnosus* | *annulatus* | AY850834 | AY850897 | -- | -- |
| *Hologymnosus* | *doliatus* | AY279612 | AY279714 | AY279818 | AY279921 |
| *Iniistius* | *aneitensis* | AY279654 | AY279757 | AY279860 | AY279963 |
| *Kyphosus* | *vaigiensis* | AY279561 | AY279664 | AY279767 | AY279870 |
| *Labrichthys* | *unilineatus* | AY279613 | AY279715 | AY279819 | AY279922 |
| *Labroides* | *bicolor* | AY279614 | AY279716 | AY279820 | AY279923 |
| *Labroides* | *dimidiatus* | AY279615 | AY279717 | AY279821 | AY279924 |
| *Labroides* | *rubrolabiatus* | AY279616 | AY279718 | AY279822 | AY279925 |
| *Labropsis* | *australis* | AY279617 | AY279719 | AY279823 | AY279926 |
| *Lachnolaimus* | *maximus* | AY279618 | AY279720 | AY279824 | AY279927 |
| *Larabicus* | *quadrilineatus* | AY279619 | AY279721 | AY279825 | AY279928 |
| *Leptojulis* | *cyanopleura* | AY279620 | AY279722 | AY279826 | AY279929 |
| *Leptoscarus* | *vaigiensis* | EU601190 | AY081094 | EU601272 | EU601319 |
| *Macropharyngodon* | *geoffroy* | AY279621 | AY279723 | AY279827 | AY279930 |
| *Macropharyngodon* | *meleagris* | AY279622 | AY279724 | AY279828 | AY279931 |
| *Malapterus* | *reticulatus* | AY279623 | AY279725 | AY279829 | AY279932 |
| *Neoodax* | *balteatus* | AY279657 | AY279760 | AY279863 | AY279966 |
| *Nicholsina* | *usta* | AY279624 | AY279726 | AY279830 | AY279933 |
| *Notolabrus* | *gymnogenis* | AY279625 | AY279727 | AY279831 | AY279934 |
| *Novaculichthys* | *macrolepidotus* | AY279626 | AY279729 | AY279832 | AY279935 |
| *Novaculichthys* | *taeniourus* | AY279627 | AY279729 | AY279833 | AY279936 |
| *Novauloides* | *macrolepidotus* | AY279626 | AY279728 | AY279832 | AY279935 |
| *Odax* | *acroptilus* | AY279658 | AY279761 | AY279864 | AY279967 |
| *Odax* | *cyanoallix* | AY279659 | AY279762 | AY279865 | AY279968 |
| *Odax* | *cyanomelas* | AY279660 | AY279763 | AY279866 | AY279969 |
| *Odax* | *pullus* | AY279661 | AY279764 | AY279867 | AY279970 |
| *Ophthalmolepis* | *lineolata* | AY279628 | AY279731 | AY279834 | AY279937 |
| *Oxycheilinus* | *bimaculatus* | AY279629 | AY279732 | AY279835 | AY279938 |
| *Oxycheilinus* | *celebicus* | AY279630 | AY279733 | AY279836 | AY279939 |
| *Oxycheilinus* | *digramma* | EU601221 | EU601258 | EU601300 | EU601350 |
| *Oxycheilinus* | *unifasciatus* | AY279631 | AY279734 | AY279837 | AY279940 |
| *Oxyjulis* | *californica* | AY279632 | AY279735 | AY279838 | AY279941 |
| *Pictilabrus* | *laticlavius* | AY279633 | AY279736 | AY279839 | AY279942 |
| *Platax* | *orbicularis* | AY279562 | AY279665 | AY279768 | AY279871 |
| *Pseudocheilinus* | *octataenia* | AY279634 | AY279737 | AY279840 | AY279943 |
| *Pseudocheilinus* | *octotaenia* | AY279634 | AY279737 | AY279840 | AY279943 |
| *Pseudocoris* | *yamashiroi* | AY279635 | AY279738 | AY279841 | AY279944 |
| *Pseudodax* | *moluccanus* | AY279636 | AY279739 | AY279842 | AY279945 |
| *Pseudodax* | *moluccanus* | EU601227 | EU601262 | EU601306 | EU601356 |
| *Pseudojuloides* | *atavai* | AY279637 | AY279740 | AY279843 | AY279946 |
| *Pseudojuloides* | *cerasinus* | AY279638 | AY279741 | AY279844 | AY279947 |
| *Pseudolabrus* | *gayi* | AY279639 | AY279742 | AY279845 | AY279948 |
| *Pteragogus* | *amboinensis* | AY279640 | AY279743 | AY279846 | AY279949 |
| *Pteragogus* | *cryptus* | AY279641 | AY279744 | AY279847 | AY279950 |
| *Ptychochromis* | *oligacanthus* | AY279564 | AY279667 | AY279770 | AY279873 |
| *Scarus* | *altipinnis* | EU601192 | EU601237 | EU601273 | EU601321 |
| *Scarus* | *chameleon* | EU601193 | EU601238 | EU601274 | EU601322 |
| *Scarus* | *coelestinus* | EU601194 | AY081084 | AY081101 | EU601323 |
| *Scarus* | *dimidiatus* | AY279642 | AY279745 | AY279848 | AY279951 |
| *Scarus* | *festivus* | EU601196 | EU601239 | EU601276 | EU601325 |
| *Scarus* | *flavipectoralis* | EU601197 | AY081086 | EU601277 | EU601326 |
| *Scarus* | *forsteni* | EU601198 | EU601240 | EU601278 | EU601327 |
| *Scarus* | *frenatus* | AY279643 | AY279746 | AY279849 | AY279952 |
| *Scarus* | *ghobban* | EU601200 | EU601241 | EU601279 | EU601329 |
| *Scarus* | *globiceps* | EU601201 | EU601242 | EU601280 | EU601330 |
| *Scarus* | *guacamaia* | EU601202 | EU601243 | EU601281 | EU601331 |
| *Scarus* | *hypselopterus* | EU601204 | EU601245 | EU601283 | EU601333 |
| *Scarus* | *iseri* | EU601203 | EU601244 | EU601282 | EU601332 |
| *Scarus* | *niger* | EU601205 | EU601246 | EU601284 | EU601334 |
| *Scarus* | *oviceps* | EU601206 | EU601247 | EU601285 | EU601335 |
| *Scarus* | *prasiognathos* | EU601207 | EU601248 | EU601286 | EU601336 |
| *Scarus* | *psittacus* | EU601208 | EU601249 | EU601287 | EU601337 |
| *Scarus* | *quoyi* | EU601210 | EU601251 | EU601289 | EU601339 |
| *Scarus* | *rivulatus* | EU601211 | EU601252 | EU601290 | EU601340 |
| *Scarus* | *rubroviolaceus* | EU601212 | EU601253 | EU601291 | EU601341 |
| *Scarus* | *schlegeli* | EU601213 | EU601254 | EU601292 | EU601342 |
| *Scarus* | *spinus* | EU601214 | EU601255 | EU601293 | EU601343 |
| *Scarus* | *taeniopterus* | EU601215 | EU601256 | EU601294 | EU601344 |
| *Scarus* | *tricolor* | EU601216 | EU601257 | EU601295 | EU601345 |
| *Semicossyphus* | *pulcher* | AY279644 | AY279747 | AY279850 | AY279953 |
| *Siphonognathus* | *argyrophanes* | AY279662 | AY279765 | AY279868 | AY279971 |
| *Sparisoma* | *aurofrenatum* | EU601217 | AY081082 | EU601296 | EU601346 |
| *Sparisoma* | *chrysopterum* | AY279645 | AY279748 | AY279851 | AY279954 |
| *Sparisoma* | *viride* | EU601219 | AY081081 | EU601298 | EU601348 |
| *Stethojulius* | *bandenensis* | AY279646 | AY279749 | AY279852 | AY279955 |
| *Stethojulius* | *trilineata* | AY279647 | AY279750 | AY279853 | AY279956 |
| *Tautoga* | *onitis* | AY279648 | AY279751 | AY279854 | AY279957 |
| *Tautogolabrus* | *adspersus* | AY279649 | AY279752 | AY279855 | AY279958 |
| *Thalassoma* | *bifasciatum* | AY279650 | AY279753 | AY279856 | AY279959 |
| *Thalassoma* | *lunare* | AY279651 | AY279754 | AY279857 | AY279960 |
| *Thorichthys* | *meeki* | AY279566 | AY279669 | AY279772 | AY279875 |
| *Wetmorella* | *nigropinnata* | AY279652 | AY279755 | AY279858 | AY279961 |
| *Xiphocheilus* | *typus* | AY279653 | AY279756 | AY279859 | AY279962 |
| *Xyrichtys* | *martinicensis* | AY279655 | AY279758 | AY279861 | AY279964 |
